# Fused Deposition Modeling (FDM) 3D Printing of the Thermo-Sensitive Peptidomimetic Drug Enalapril Maleate

**DOI:** 10.3390/pharmaceutics14112411

**Published:** 2022-11-08

**Authors:** Lena Hoffmann, Jörg Breitkreutz, Julian Quodbach

**Affiliations:** 1 Institute of Pharmaceutics and Biopharmaceutics, Heinrich Heine University, Universitätsstraße 1, 40225 Düsseldorf, Germany; 2 Department of Pharmaceutics, Utrecht University, Universiteitsweg 99, 3584 CG Utrecht, The Netherlands

**Keywords:** hot-melt extrusion, FDM 3D printing, thermo-sensitive drug, decomposition, analytics of extrudates and 3D printed tablets, oral dosage form, personalized medicine

## Abstract

Fused deposition modeling (FDM) 3D printing was used to produce 3D printed tablets with the thermo-sensitive model peptidomimetic drug enalapril maleate (EM). Two different formulations were prepared to investigate the degradation of enalapril maleate during the FDM 3D printing process. Soluplus^®^ and Eudragit^®^ E PO were chosen as polymers. After hot-melt extrusion (HME) and FDM 3D printing, both formulations were characterised regarding their solid-state properties using DSC and XRD. The degradation of the drug was analysed by determination of the content in the extrudates and 3D printed tablets, and dissolution was assessed. Various approaches have been attempted to prevent degradation of enalapril maleate, including utilization of a larger nozzle diameter and higher printing speeds to reduce heat exposition. None of these approaches were successful in preventing drug degradation. However, significant differences in the amount of degradation between the two formulations with different polymers could be observed. Thus, the FDM 3D printing process was not feasible without any degradation for the thermo-sensitive drug enalapril maleate. A maximum of 85.55 ± 1.48% enalapril was recovered in Eudragit^®^ E PO tablets printed with a 0.4 mm nozzle at a temperature of 180 °C and with a speed of 30 mm/s.

## 1. Introduction

Nowadays, personalized medicine is gaining increasing interest in the health care sector. The conventional drug treatment approach is based on a “one size fits all” principle where most patients receive the same drug at the same dosages and administration frequencies [1]. This can lead to varying responses and can also be associated with adverse drug reactions [1]. In personalized medicine, medical treatments are tailored to patients based on genetic profile, concurrent medicines, and disease state [2,3]. The most promising technology that can drive the paradigm shift to personalized medicine is 3D printing [4]. 3D printing provides the possibility of producing 3D objects layer by layer with the aid of appropriate computer software [4]. 3D printing technologies offer a broad range of applications in the pharmaceutical field. Binder Jetting (BJ), Stereolithography (SLA), Selective Laser Sintering (SLS), Semi-solid extrusion (SSE) and Fused Deposition Modeling (FDM) are researched for pharmaceutical applications [5,6]. The production of 3D printed dosage forms offers several advantages compared to the conventional manufacturing process which includes, for example, the following aspects: customization, polypharmacy, flexible design, point of care production, and waste minimization [7]. 3D printing affords us the possibility of precisely producing customised dosage forms for different patient groups, including pediatric and geriatric patients and also patients with pharmacogenetic polymorphism. Furthermore, it can address the needs of these patient groups in terms of swallowability and taste. Besides other benefits, 3D printing can also be considered environmentally friendly due to low amounts of waste [7].

**Figure 1:**
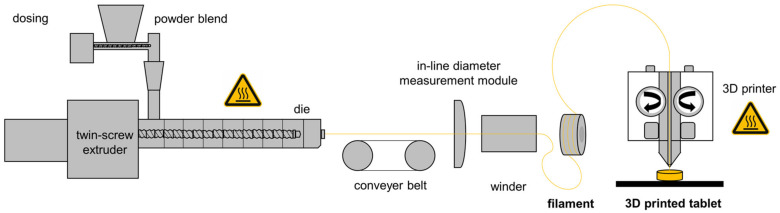
Illustration of the two heat-intensive processes for the manufacturing of 3D printed tablets by HME and FDM (modified from [9]).

Among the above-mentioned 3D printing techniques, FDM 3D printing is the most widely explored technology for the production of patient-specific dosage forms due to the relatively straightforward, solvent free procedure, its versatility and speed, and also the inexpensive equipment [7,8,9]. Prefabricated extrudates processed by hot-melt extrusion serve as feedstock material for the 3D printing process (Figure 1) [5,10].

Previous publications have demonstrated that thermally stable drugs could be extruded into extrudates with different polymers and processed into 3D printed dosage forms using FDM 3D printers. Due to the increased interest in 3D printed dosage forms, the behavior of thermo-sensitive drugs, including biological agents, during the 3D printing process warrants attention [7]. The world health organization (WHO) defines a time- and temperature-sensitive pharmaceutical product (TTSPP) as “Any pharmaceutical good or product which, when not stored or transported within predefined environmental conditions and/or within predefined time limits, is degraded to the extent that it no longer performs as originally intended” [11]. Although this definition usually refers to the storage in a refrigerator, Pazo-Oubiña et al. (2021) stated that this definition can also be applied to drugs which degrade during the 3D printing process due to the high printing temperature or UV light/beams [12].

Reduction in hot-melt extrusion and 3D printing temperatures is the most promising approach to reduce drug degradation. So far, the following efforts have been made on the fabrication of 3D printed tablets at lower temperatures. Okwuosa et al. (2016) produced patient-specific immediate release tablets with either the active ingredient theophylline or dipyridamole. Besides the active ingredients, the polymer polyvinylpyrrolidone (PVP), the plasticizer triethyl citrate, and the filler talc were used to achieve a lower FDM 3D printing at a temperature of 110 °C [13]. Kempin et al. (2018) also printed immediate release tablets with the model drug pantoprazole sodium at temperatures below 100 °C. Five different pharmaceutical grade polymers, poly vinylpyrrolidone (PVP K12), polyethylene glycol 6000 (PEG 6000), Kollidon^®^ VA 64, polyethylene glycol 20,000 (PEG 20,000) and poloxamer 407, were hot-melt extruded and then printed to tablets at lower temperatures [14]. Kollamaram et al. (2018) investigated the two immediate release polymers, Kollidon^®^ VA 64 and Kollidon^®^ 12 PF, for the FDM 3D printing of ramipril at lower temperatures. It could be extruded at 70 °C and printed into tablets at a temperature of 90 °C. Kollidon^®^ VA 64 and Kollidon^®^ 12 PF were also demonstrated to be suitable for printing the drug 4-aminosalicylic acid (4-ASA), which shows degradation in FDM 3D printing in previous studies [15]. Manini et al. (2022) created long-acting implantable dosage forms with the heat-sensitive drug paliperidone palmitate. Different formulations consisting of polylactic acid (PLA) and poloxamer 188, PLA and PEG 2000 or PLA and ethylene-vinyl acetate (EVA) were extruded at 180 °C and printed at 150 °C [6]. Sadia et al. (2018) produced 3D printed bilayer antihypertensive tablets with the active ingredient hydrochlorothiazide and enalapril maleate and combined both in a single bilayer tablet by using a dual FDM 3D printer [16].

For the present study, we selected enalapril maleate, an angiotensin-converting enzyme inhibitor (ACEI), as the model drug for the investigations of thermal stress during the 3D printing process. Enalapril maleate has a peptidomimetic structure and is therefore highly thermo-sensitive. Additionally, antihypertensive substances such as enalapril maleate are of particular importance for the use in personalized medicine because a slow titration of the dosage is frequently necessary [17]. Antihypertensives are among the most commonly used drugs in 3D printing formulations, along with anti-inflammatory and analgesic drugs [18]. Regarding the therapeutic regimen for the active ingredient enalapril maleate, the dosage range for the treatment of hypertension in adults is between 2.5 mg and 40 mg. For children, the dose should be adjusted to the body weight [17].

In a previous publication, suitable process conditions and formulations with the polymers Soluplus^®^ and Eudragit^®^ E PO were investigated for hot-melt extrusion of enalapril maleate. At certain suitable process conditions, it showed minimal to no degradation of the active ingredient compared to other polymers. An interaction between the drug and Eudragit^®^ E PO led to stabilization of the drug [19].

Therefore, the aim of this work is to investigate the consecutive drug printing of the HME filaments via FDM. The nozzle diameter, the printing temperature, and the printing speed were examined as factors to keep thermal degradation of the peptidomimetic drug enalapril maleate as minimal as possible.

## 2. Materials and Methods

### 2.1. Materials

Enalapril maleate was purchased from Azelis (Sankt Augustin, Germany) and manufactured by Zhejiang Huahai Pharmaceutical Industry (Taizhou, China). Polyethylene oxide (PEO) Mw 100,000 (POLYOX™ WSR N10) was kindly provided by DuPont Nutrition & Biosciences (Neu-Isenburg, Germany). Soluplus^®^ (SOL) was kindly provided by BASF (Ludwigshafen, Germany). Basic butylated methacrylate copolymer (bPMMA, Eudragit^®^ E PO) and fumed silica (SiO_2_, Aerosil^®^ 200 *v*/*v* Pharma) were kindly provided by Evonik (Essen, Germany). Enalapril maleate United States Pharmacopoeia (USP) Reference Standard were purchased by Eurofins PHAST GmbH (Homburg, Germany). Enalapril diketopiperazine (DKP) was purchased from LGC Standards GmbH (Wesel, Germany). Other chemicals were of reagent grade.

### 2.2. Preparation of Drug Loaded Extrudates

Hot-melt extrusion was carried out in our previous paper for the production of extrudates with the preferred formulations with a drug load of 10% enalapril maleate at a reduced temperature of 100 °C and additionally, for formulation, F2 at 70 °C (Table 1) [19]. HME was repeated for formulation F2 at 70 °C as the lowest possible extrusion temperature for the production of a sufficient number of 3D printed tablets for the printing study.

Briefly, a Leistritz ZSE 12 extruder was used with either a ZD 5 FB gravimetric feeder (Three-Tec, Seon, Switzerland) or a volumetric feeder (Brabender MT-S-HYD, Brabender, Duisburg, Germany). Both formulations were dosed with a feed rate of 100 g/h.

### 2.3. 3D Printing of Enalapril Maleate Tablets

The drug-loaded extrudates used as a substrate for FDM printing were printed into tablets using an i3 Mk3 printer (Prusa Research; Czech Republic). The tablet geometry was designed using Autodesk Fusion 360 (Mill Valley, CA, USA) and then sliced using the PrusaSlicer (PRUSA Research; Hradec Králové, Czech Republic). The extrudates were printed with two different nozzle diameters of 0.4 mm and 0.6 mm at nozzle temperatures of 180 °C and 190 °C and a print bed temperature of 35 °C with printing speeds of 30 mm/s, 60 mm/s, and 90 mm/s. A cylindrical geometry (diameter: 6.8 mm and height: 2.4 mm) was selected to obtain tablets with a dose of 10 mg with a drug loading of 10% enalapril maleate in the extrudates.

### 2.4. Scanning Electron Microscopy (SEM) Imaging

Morphology of extrudates and 3D printed tablets of formulation F2 based on bPMMA extruded at 70 °C was examined using Zeiss Scanning Electron Microscope Leo 1430 VP (Zeiss, Jena, Germany). Samples were sputtered with a thin gold layer. The measurements were completed under a vacuum at a working voltage of 5–10 kV. The surface of the extrudates and tablets, as well as the surface side view of the 3D printed tablets, were visualized.

### 2.5. Solid State Analysis

#### 2.5.1. DSC Analysis

Thermo analysis of starting materials, physical mixtures as well as extrudates and tablets, was performed using differential scanning calorimetry (DSC) (DSC 1, Mettler-Toledo, Giessen, Germany). Samples were heated at 10 °C/min over the temperature range of 20 °C to 200 °C for the active ingredient enalapril maleate and of 20 °C to 160 °C for the physical mixtures, extrudates, and tablets.

#### 2.5.2. X-ray Diffractometer (XRD)

XRD analysis was carried out for the starting materials, extrudates, and tablets using a Rigaku Miniflex diffractometer in Θ/2Θ geometry at an ambient temperature using Cu-Kα radiation (Θ = 1.54182 Å). Scans were performed from 2θ = 2° to 50° in 0.01° steps.

### 2.6. Drug Content of Extrudates and 3D Printed Tablets

A 3D printed tablet or a section of a drug-loaded extrudate (approximately 0.1 g) was placed in a 100 mL volumetric flask and dissolved in 100 mL of a mixture of acetonitrile and 1 mM potassium dihydrogen phosphate buffer (50/50, *v*/*v*). Samples of the solutions were then filtered through a 0.45 μm nylon filter. An Elite LaChrom system consisting of L-2200 automatic sampler, L-2130 high pressure pump, L-2300 column oven, and L-2400 UV detector was used (all Hitachi-VWR, Darmstadt, Germany). Chromatographic separation of enalapril maleate and possible degradation products were carried out on a XBridge C18 column [3.0 × 150 mm, 3.5 µm (Waters, Eschborn, Germany)]. After injection of 30 µL sample solution, the samples were separated with the aid of a gradient using a mobile phase of acetonitrile and 1 mM potassium dihydrogen phosphate buffer pH 3.0 (Table 2). The flow rate was 1.0 mL/min and the column temperature was maintained at 65 °C. The eluent was screened at a wavelength of 215 nm. The run time was set to 15 min. n = 10 samples were measured and mean ± SD are shown in the discussion. As described in [19], the identification and quantification of enalapril maleate and related substances was ensured with the help of reference standards. Selectivity was achieved by spiking enalapril maleate CRS solution with the other related substances. The limit of detection (LOD) was determined to be 19.4 ng/mL and the limit of quantification (LOQ) to be 58.7 ng/mL. Linearity was demonstrated in each case with an R^2^ > 0.999 for the content uniformity in a concentration range from 60 to 140 µg/mL and for the dissolution study in a concentration range of 0.2 to 12 µg/mL. The accuracy of the content determination in the samples was verified by using enalapril maleate USP reference standard and enalapril diketopiperazine standard with known contents. The determination of the content of enalapril diketopiperazine was completed by external calibration and was freshly prepared for the analyses. Precision, determined as repeatability, for enalapril was ensured with a coefficient of variation (CV) of 0.74%.

### 2.7. Dissolution

An in vitro drug release study was carried out using USP type I apparatus (AT7 Smart, Sotax, Aesch, Switzerland) at 37 °C ± 0.5 °C with a rotating speed of 50 rpm in 900 mL 0.1 N hydrochloric acid (n = 3, mean ± SD). The amount of ENP and DKP was quantified by HPLC.

## 3. Results

### 3.1. Hot-Melt Extrusion and 3D Printing of Tablets

A thorough discussion of the formulation composition and adjustment of the critical process parameters of the extrusion process can be found in our previous paper [19]. The obtained extrudates from formulations made from SOL and bPMMA are looking flexible for the subsequent 3D printing process. Cylindrical tablets were printed at nozzle temperatures of 180 °C and 190 °C and a print bed temperature of 35 °C.

### 3.2. Scanning Electron Microscopy (SEM) Imaging

**Figure 2:**
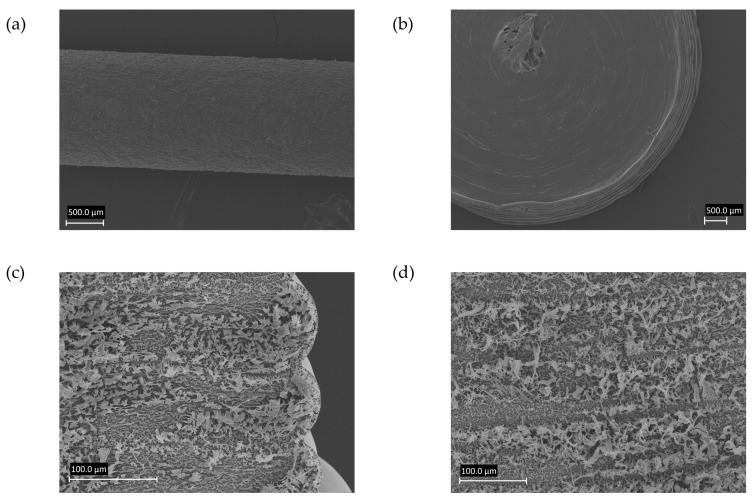
SEM images of the extrudate surface (**a**), tablet surface (**b**), and tablet side view (**c**,**d**) of the formulation containing bPMMA extruded at 70 °C.

SEM images of formulation F2 extruded at 70 °C are shown in Figure 2a,b and display the surface of an extrudate and a 3D printed tablet. The layer-by-layer structure and the texture of the 3D printed tablet can be seen in Figure 2c,d. Tidau et al. (2019) investigated the surface of PEO-based extrudates and described the surface appearance as an octagonal structuring [20]. This structuring indicated the crystalline part of the semi-crystalline polymer PEO of which the crystal structure was investigated by Takahashi et al. (1973) [21].

### 3.3. Solid State Analysis

#### 3.3.1. Thermo Analysis

**Figure 3:**
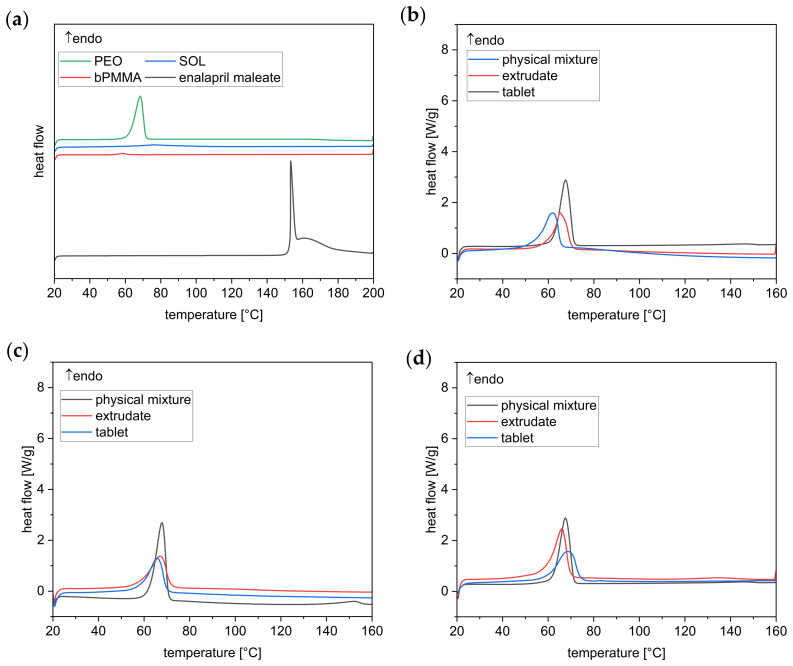
DSC thermograms of the starting materials (**a**), the formulation containing SOL extruded at 100 °C (**b**), the formulation containing bPMMA extruded at 100 °C (**c**), and at 70 °C (**d**), respectively.

Differential scanning calorimetry (DSC) analysis of enalapril maleate starting material and physical mixtures showed an endothermic peak at 153 °C (Figure 3a) [22,23]. However, the peak was not present in the extrudates and 3D printed tablets of the two different formulations, indicating the absence of crystalline enalapril maleate. Therefore, enalapril maleate or its degradation product seems to be in an amorphous state in the extrudates and 3D printed tablets [16]. The semi-crystalline PEO also depicts an endothermic event with an onset at approximately 62 °C and a peak at 68 °C, which can be seen in the physical mixture, extrudates, and tablets (Figure 3b–d) [20].

#### 3.3.2. X-ray Diffractometer (XRD)

In addition to the DSC analysis, XRD analysis was performed to investigate changes in the crystallinity of enalapril maleate starting material and the physical mixtures in comparison to the extrudates and 3D printed tablets for both formulations.

**Figure 4:**
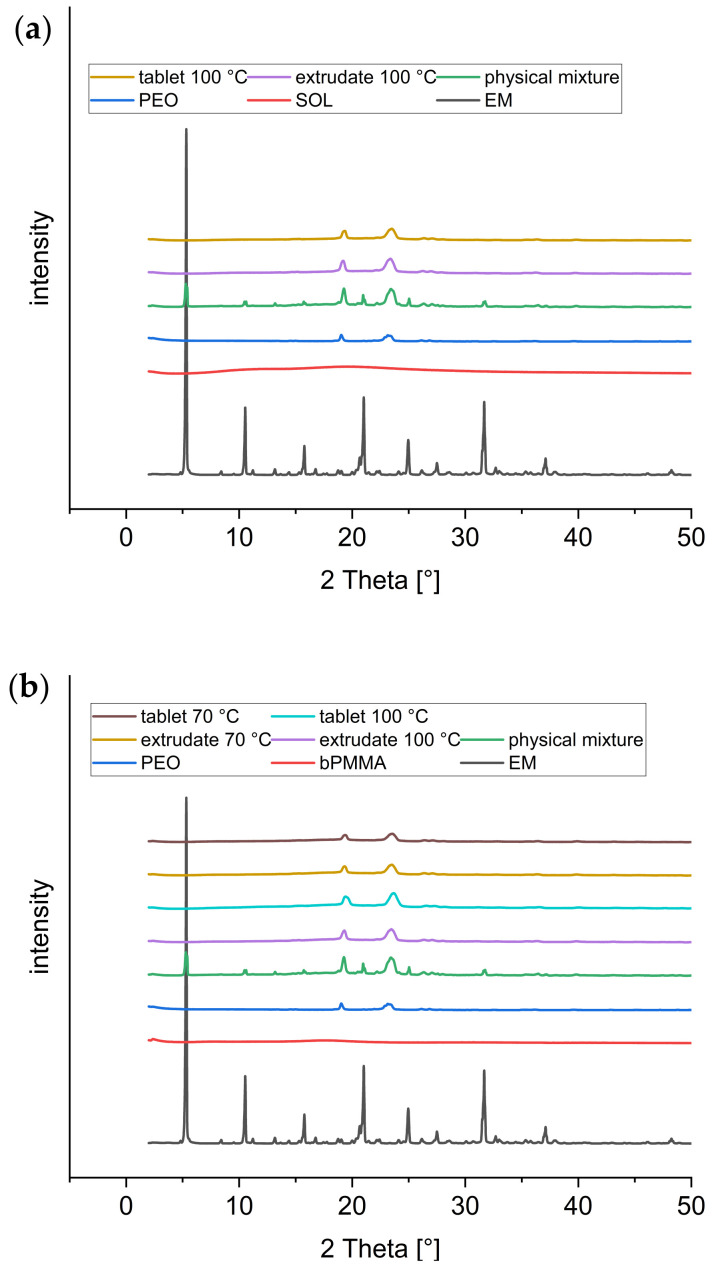
X-ray diffractograms of the formulation containing SOL extruded at 100 °C (**a**) and the formulation containing bPMMA extruded at 100 °C and 70 °C (**b**), respectively.

Enalapril maleate showed a crystalline structure with characteristic peaks at 5.3°, 8.1°, 10.5°, 15.7°, 21.5°, 24.9°, and 31.6° as previously described by Ramírez-Rigo et al. (2014) [24] (Figure 4a,b). For the polymers SOL (Figure 4a) and bPMMA (Figure 4b), no peaks were present due to the amorphous nature of these polymers. Polyethylene oxide exhibited two diffraction peaks at 19.0° and 23.2° (Figure 4a,b) [25]. In the physical mixtures of both formulations, the characteristic peaks from enalapril maleate could be observed. The examined extrudates and 3D printed tablets revealed no crystalline active ingredient, only the crystalline parts of PEO were visible in the diffractograms. Furthermore, no differences in the physical state were found between the two formulations with SOL (Figure 4a) and bPMMA (Figure 4b) after extrusion at 100 °C [19] and FDM 3D printing at 180 °C. Reducing the extrusion temperature of the bPMMA formulation to 70 °C was also sufficient to fully convert enalapril maleate to the amorphous state (Figure 4b).

DSC and XRD measurements both demonstrate that enalapril maleate is in amorphous form in the extrudates and 3D printed tablets of both formulations.

### 3.4. Drug Content of Extrudates and 3D Printed Tablets

**Figure 5:**
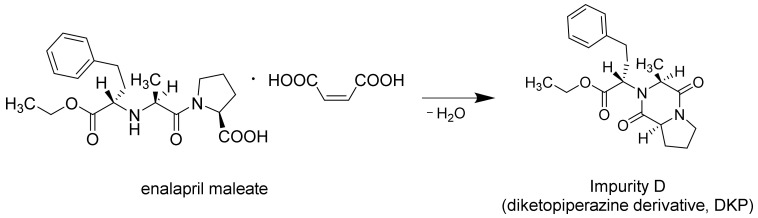
Illustration of the conversion of enalapril maleate to Impurity D at elevated temperatures.

The content of enalapril and degradation products in extrudates and 3D printed tablets were analysed as described in Section 2.6. The diketopiperazine derivative (DKP, Impurity D), which is described as the intramolecular cyclization product of enalapril [19], was found to be the main and only degradation product in both the extrudates and the 3D printed tablets of the formulations (Figure 5).

**Figure 6:**
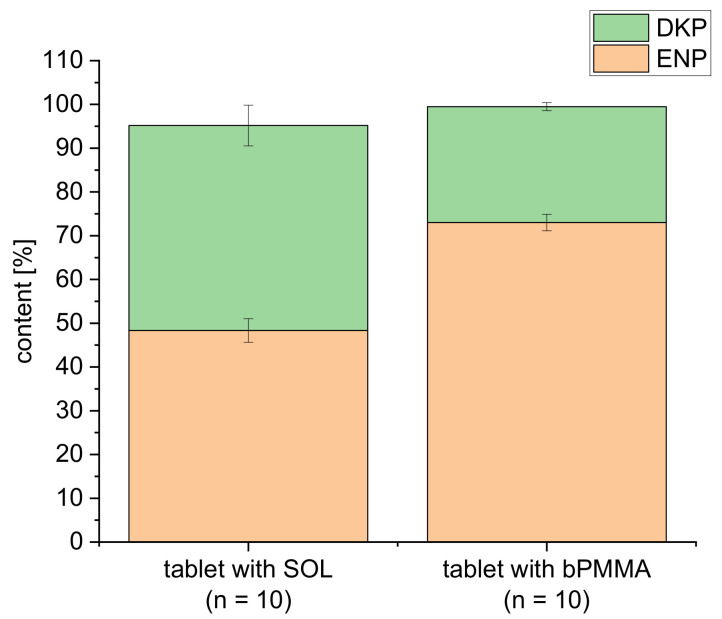
Illustration of the enalapril and diketopiperazine derivative contents after 3D printing for the formulation containing SOL and the formulation containing bPMMA extruded at 100 °C and printed at 180 °C (n = 10, mean ± SD).

Content data after extrusion have been discussed in our paper [19]. For formulation F1, it was observed that the content of enalapril in the extrudates extruded at 100 °C was 94.83 ± 1.72% after extrusion [19] and decreased after the FDM 3D printing process to 48.34 ± 2.69% (Figure 6). DKP was formed during both heat-intensive processes. A content of 7.54 ± 0.24% DKP was determined in the extrudates [19] and 46.84 ± 4.65% in the 3D printed tablets (Figure 6).

For formulation F2 extruded at a temperature of 100 °C, the content of enalapril was 98.44 ± 0.30% [19]. After 3D printing, the content of enalapril decreased to 73.01 ± 1.91% (Figure 6). Again, the degradation of enalapril is accompanied by an increase in the content of the DKP. A total of 2.83 ± 0.09% DKP was found in the extrudates [19], whereas 26.48 ± 0.92% DKP was formed during the FDM 3D printing process *(*Figure 6).

**Figure 7:**
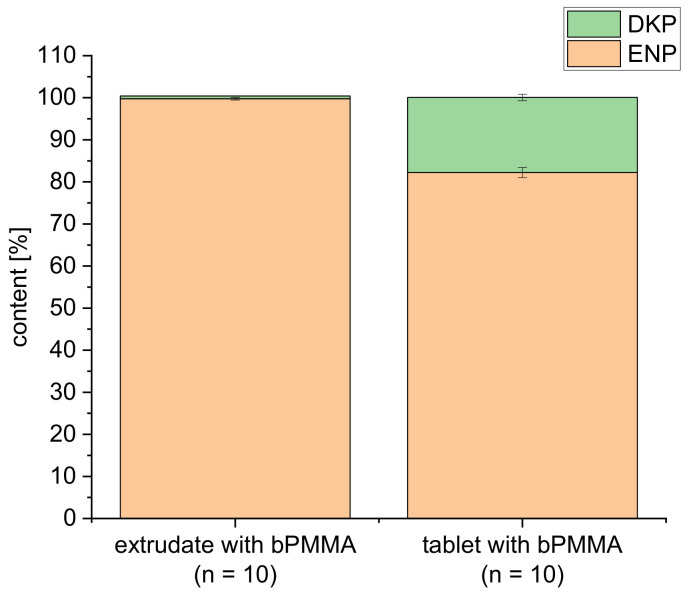
Illustration of the enalapril and diketopiperazine derivative contents after extrusion and 3D printing for the formulation containing bPMMA extruded at 70 °C and printed at 180 °C (n = 10, mean ± SD).

Comparing both formulations, a higher drug content was found for formulation F2 with the polymer bPMMA under the same process conditions during hot-melt extrusion and FDM 3D printing. This could be explained with the stabilizing effect of the basic polymer bPMMA in comparison to the neutral polymer SOL as described in [19]. This demonstrates the impact formulation constituents may have on the degradation of drugs. Formulation F2, which was also extruded at a temperature of 70 °C and repeated again, revealed an enalapril content of 99.75 ± 0.32% compared to 101.72 ± 1.65% as already shown in our previous paper [19]. After 3D printing, 82.21 ± 1.21% enalapril was recovered (Figure 7). A small amount with 0.68 ± 0.03% DKP was found in the extrudates and 17.86 ± 0.77% in the 3D printed tablets. Thus, FDM 3D printing at 180 °C had the main influence on the degradation of the active ingredient. However, printing at lower temperatures was not possible, most likely due to high melt viscosity of both formulations.

Different strategies to reduce degradation were investigated. Tablets were originally printed at a printing speed of 30 mm/s and higher printing speeds were used to investigate if shorter residence times had an influence on degradation, as an exponential decrease in printing time with higher printing speed has been reported [26]. A larger nozzle diameter should enable higher material flow while reducing shear forces, potentially resulting in shorter residence times and less degradation. Tablets were also printed at a higher nozzle temperature of 190 °C to investigate if decreased melt viscosity would allow for higher printing speeds and shorten the residence time. Lastly, it was investigated, if lower infill levels enabled quicker cooling and reduced degradation.

Therefore, ten tablets were printed with nozzle diameters of 0.4 mm and 0.6 mm at temperatures of 180 °C and 190 °C and 35 °C print bed temperature with printing speeds of 30 mm/s, 60 mm/s, and 90 mm/s, and the content of enalapril and the diketopiperazine derivative was determined.

Surprisingly, a slight decrease in enalapril content was observed with a nozzle diameter of 0.4 mm and increasing printing speed. This was observed at printing temperatures of 180 °C and 190 °C (Table 3). At 180 °C and a printing speed of 30 mm/s, 85.55 ± 1.48% enalapril was recovered; whereas, the content of enalapril at the highest printing speed of 90 mm/s was 79.97 ± 1.80%. Tablets printed at 190 °C and 30 mm/s had a drug content of 78.47 ± 0.77%. At a printing speed of 90 mm/s, only 74.98 ± 1.47% enalapril was found. The decrease in the content of enalapril for the 0.4 mm nozzle at higher printing speeds may be explained with increased back pressure during 3D printing at the same temperature, which might lead to more back-mixing. Furthermore, it could be observed that a higher nozzle temperature always led to a higher degradation of enalapril in the 3D printed tablets (Table 3).

Using a nozzle diameter of 0.6 mm resulted in a small increase in enalapril content at a printing temperature of 180 °C with increasing printing speed (Table 4). A total of 79.40 ± 0.64% enalapril was found in the 3D printed tablets at 30 mm/s and 84.41 ± 1.30% at 90 mm/s. This could be explained due to a shorter residence time and heat exposure during printing. At 190 °C, 79.27 ± 1.36% enalapril was found at 30 mm/s and 79.24 ± 0.98% at 90 mm/s. No increase in the content could be observed at a temperature of 190 °C. At a printing speed of 60 mm/s, a lower content of 75.32 ± 1.53% was observed. Comparable to printing with the 0.4 mm nozzle, a lower content was found for all printing speeds at a higher temperature.

F- and *t*-tests (α = 0.05) were performed for recovery rates of enalapril printed at 30 mm/s and 90 mm/s to explore whether differences were significant at both temperatures, both nozzle diameters, and between the highest printing speed at 190 °C. The analysis showed that for the 0.4 mm nozzle, there were significant differences in the content between the printing speeds 30 mm/s and 90 mm/s at both temperatures. For the 0.6 mm nozzle, the differences were significantly different only at a temperature of 180 °C. A comparison between the two nozzles for the highest printing speed of 90 mm/s also showed significant differences.

In summary, the investigations into the influence of the nozzle diameter and the printing speed showed that the highest amount of enalapril was found for the 0.4 mm nozzle at a nozzle temperature of 180 °C and a printing speed of 30 mm/s (85.55 ± 1.48%). Nonetheless, utilizing a larger nozzle diameter of 0.6 mm and a printing speed of 90 mm/s, printing times were reduced without sacrificing accuracy, as demonstrated by the not significantly lower but uniform drug content of 84.41 ± 1.30%.

**Figure 8:**
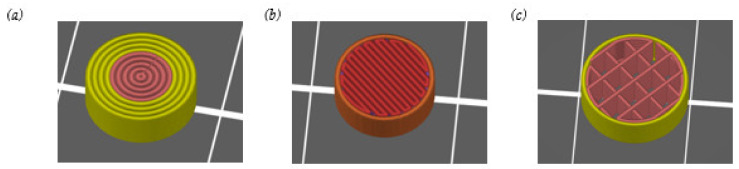
Design of different 3D printed tablets of the formulation containing bPMMA extruded at 70 °C with 100 % infill (**a**), and reduced infill of 25 % (**b**) and 20 % (**c**).

In addition to 3D printing of tablets with 100% infill, the influence of reduced infill and other contours on the content of enalapril should also be investigated. Tablets were printed with a nozzle diameter of 0.4 mm. Therefore, besides the cylindrical tablets with 100% infill (Figure 8a), tablets were printed with reduced infill (25% and 20%) and only one contour (n = 3, mean ± SD) (Figure 8b,c). Infills appear to deviate more than 5% in Figure 8 because of the visualization in the slicer software.

With another design (Figure 8b), the content of enalapril was 78.20 ± 0.42% (n = 3, mean ± SD) and the content of the diketopiperazine derivative was 22.74 ± 0.54% (n = 3, mean ± SD) in the tablets. This did not show a higher content of enalapril despite reduced infill. The infill was further reduced to 20% and straight infill was chosen (Figure 8c). To achieve the same tablet weight of 100 mg, the tablet geometry had to be adjusted to a diameter of 9.6 mm and a height of 3.4 mm. The content of enalapril in these tablets was 81.93 ± 2.85% (n = 3, mean ± SD), whereas 19.27 ± 2.33% (n = 3, mean ± SD) diketopiperazine derivative was formed.

Compared to the 3D-printed tablets with 100% infill, no higher content has been found by printing more flexible structures with reduced infill or varying infill pattern.

### 3.5. Dissolution

**Figure 9:**
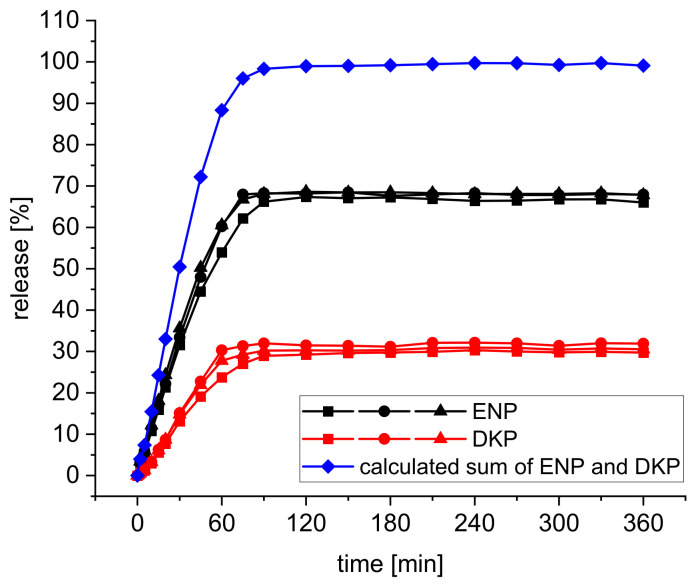
In vitro dissolution profiles of ENP and DKP from bPMMA tablets (n = 3), as well as the calculated sum of ENP and DKP, pH 1.2 buffer solution, USP Apparatus 1-basket, 37 °C, 50 rpm.

In addition to the content, the release behavior of enalapril and DKP (Figure 9) from the bPMMA tablets extruded at 70 °C with 100% infill was also investigated compared to the extrudates which was already shown in [19]. As expected, fast drug release was observed. Due to the decrease in enalapril content in the tablets and the formation of the DKP, only 35.62 ± 5.52% enalapril and 14.78 ± 1.52% DKP were dissolved in the tablets after 30 min [27]. After 180 min, 68.85 ± 1.86% enalapril and 30.34 ± 0.72% DKP were released. However, despite the degradation of enalapril in the tablets, enalapril was almost completely released from the tablets, which is shown by the formation of the calculated sum of ENP and DKP (Figure 9). The sum, which was formed from the mean values of the individual curves of both substances, amounts to 99.18% after 180 min.

## 4. Conclusions

In this study, we investigated the thermal degradation of the peptidomimetic drug enalapril maleate in FDM 3D printing in two different formulations. Data showed that enalapril maleate was amorphous in extrudates and tablets of both formulations. Processed at 100 °C, a higher content of enalapril was observed in bPMMA-based extrudates compared to SOL-based extrudates. The same order was observed in tablets 3D printed at 180 °C, but the observed difference was larger. Enalapril maleate degraded more strongly during the 3D printing process in the formulation with SOL than in the formulation with bPMMA. The main thermal degradation product found in extrudates and 3D printed tablets was the diketopiperazine derivative (Imp-D). The lower degradation of enalapril maleate with bPMMA could be explained by a cation–anion interaction of enalapril with the basic bPMMA [19]. Degradation in extrudates could be avoided using bPMMA at an extrusion temperature of 70 °C. In FDM, a higher temperature gradient between nozzle and filament is necessary to plasticise the filament in the comparably short residence time, and degradation was observed in all experiments. Selecting a larger nozzle diameter and higher printing speeds as well as varying the infill did not result in reduced degradation of the drug in the 3D printed tablets.

To our knowledge, this is the first study that not only investigates the extent of degradation of a thermo-sensitive drug during 3D printing, but also quantifies both the drug and the resulting degradation product. This study revealed that despite selection of suitable polymers, FDM 3D printing with a commercial 3D printer is not feasible for the peptidomimetic enalapril maleate without degradation. Our study highlights one of the drawbacks of FDM 3D printing, as it excludes therapeutically important active ingredients such as enalapril maleate, which is especially important for pediatric care. It also demonstrates the need for the development and implementation of good manufacturing practice (GMP) compliant FDM 3D printers, designed to reduce the temperature stress during 3D printing. These could offer new opportunities for this important class of drugs. Enabling 3D printing with such drugs would be beneficial for personalized treatments.

## Figures and Tables

**Table 1:** Formulation composition for the production of HME extrudates.

Formulations	API (%)		Matrix (%)		Plasticizer (%)		Glidant (%)	
F1	EM	10	SOL	44.75	PEO	44.75	SiO_2_	0.5
F2	EM	10	bPMMA	44	PEO	44	SiO_2_	2.0

**Table 2:** Gradient for the separation of enalapril and related substances.

Time (min)	Acetonitrile (% *v*/*v*)	Buffer (% *v*/*v*)
0–1.0	2	98
1.0–1.2	2 → 25	98 → 75
1.2–5.0	25	75
5.0–7.5	25 → 40	75 → 60
7.5–9.0	40 → 75	60 → 25
9.0–11.0	75 → 95	25 → 5
11.0–12.5	95	5
12.5–12.6	95 → 2	5 → 98
12.6–15.0	2	98

**Table 3:** Enalapril (ENP) and diketopiperazine derivative (DKP) contents in the FDM printed tablets with a nozzle diameter of 0.4 mm printed at 180 °C (a) and 190 °C (b) with different printing speeds (n = 10, mean ± SD).

Temperature [°C]	Printing Speed [mm/s]					
	30		60		90	
	ENP	DKP	ENP	DKP	ENP	DKP
180	85.55 ± 1.48%	15.55 ± 0.87%	82.83 ± 1.58%	16.33 ± 0.94%	79.97 ± 1.80%	19.89 ± 1.12%
190	78.47 ± 0.77%	21.55 ± 0.75%	77.02 ± 0.89%	22.11 ± 0.59%	74.98 ± 1.47%	24.37 ± 0.99%

**Table 4:** Enalapril (ENP) and diketopiperazine derivative (DKP) contents in the FDM printed tablets with a nozzle diameter of 0.6 mm printed at 180 °C (a) and 190 °C (b) with different printing speeds (n = 10, mean ± SD).

Temperature [°C]	Printing Speed [mm/s]					
	30		60		90	
	ENP	DKP	ENP	DKP	ENP	DKP
180	79.40 ± 0.64%	18.39 ± 0.54%	79.91 ± 0.81%	18.69 ± 0.60%	84.41 ± 1.30%	15.10 ± 0.71%
190	79.27 ± 1.36%	21.43 ± 0.66%	75.32 ± 1.53%	24.66 ± 1.28%	79.24 ± 0.98%	21.63 ± 0.54%

## Data Availability

The data presented in this study are available upon request from the corresponding author.

## Abbreviations

ACEI	angiotensin-converting enzyme inhibitor
BJ	binder jetting
bPMMA	basic butylated methacrylate copolymer
CV	coefficient of variation
DKP	diketopiperazine derivative
DSC	Differential Scanning Calorimetry
EM	enalapril maleate
ENP	enalapril
EVA	ethylene-vinyl acetate
HME	hot melt extrusion
HPLC	High Performance Liquid Chromatography
FDM	fused deposition modeling
GMP	good manufacturing practice
Imp	impurity
LOD	limit of detection
LOQ	limit of quantification
PEG	polyethylene glycol
PEO	polyethylene oxide
PF	Pyrogen-free
PLA	polylactic acid
PVP	polyvinylpyrrolidone
SD	standard deviation
SEM	Scanning Electron Microscopy
SiO_2_	fumed silica
SLA	stereolithography
SLS	selective laser sintering
SOL	Soluplus
SSE	semi-solid extrusion
TTSPP	time- and temperature-sensitive pharmaceutical product
USP	United States Pharmacopeia
UV	ultraviolet
VA	poly(vinylpyrrolidone-vinyl acetate)-copolymer
WHO	World Health Organization
XRD	X-ray diffractometer
